# Positive Youth Development and Depression: An Examination of Gender Differences in Croatia and Spain

**DOI:** 10.3389/fpsyg.2021.689354

**Published:** 2022-01-21

**Authors:** Diego Gomez-Baya, Ana Babić Čikeš, Marina Hirnstein, Ana Kurtović, Gabrijela Vrdoljak, Nora Wiium

**Affiliations:** ^1^Department of Social, Developmental and Educational Psychology, Faculty of Education, Psychology and Sport Sciences, University of Huelva, Huelva, Spain; ^2^Department of Psychology, Faculty of Humanities and Social Sciences, Josip Juraj Strossmayer University of Osijek, Osijek, Croatia; ^3^Department of Psychosocial Science, Faculty of Psychology, University of Bergen, Bergen, Norway

**Keywords:** positive youth development (PYD), depression, gender, Croatia, Spain, 5Cs, cross-national

## Abstract

Depression is a major public health issue and the literature has consistently showed that the rates of depression increase dramatically during youth transition to adulthood, and gender differences merge in this period. Positive youth development (PYD) framework is focused on strengths that make young people more resistant to negative outcomes, like depression, and more capable to choose a positive life direction. The aim of the study was to examine the relationship between the 5Cs of PYD and depression in Croatia and Spain, as well as to analyze gender differences. This research was conducted within the PYD Cross-National Project. High school and university students from Eastern Croatia and Southern Spain participated in the study (*M*_age_ = 19.37, *SD* = 2.11; 62.3% female). There were 584 students from Croatia and 768 students from Spain, who filled in self-reports of PYD and depression (i.e., PYD-SF and PHQ-9, respectively). Results showed that male participants presented more Competence and Confidence, while female participants reported more Connection, Caring, Character, overall PYD, but also more depressive symptoms. Furthermore, Confidence and Connection were negative correlates of depressive symptoms, with neither Spain nor Croatia showing remarkable gender differences. These results may have some implications concerning the promotion of the 5Cs of PYD as a recommendable approach to promote youth mental health in Croatia and Spain from a gender perspective. Youth mental health services and initiatives that engage the partnership of youth contexts, such as the family, schools and neighborhoods, should not only address risk factors for mental health problems, but also protecting factors as the 5Cs, thus providing a more inclusive and sustainable support for youth well-being.

## Introduction

Previous literature has showed that depression is a major public health issue (GBD 2017 Disease and Injury Incidence and Prevalence Collaborators, 2018). According to the World Health Organization’s Global Burden of Disease project in 2001, “depression alone causes over 12% of the years lived with disability globally, and ranks as the third leading contributor to the global burden of disease (…) The average annual costs, including medical, pharmaceutical and disability costs, for employees with depression are estimated to be up to 4.2 times higher than costs for people with other conditions” (European Commission, 2004). Studies show that the rates of depression increase dramatically with the beginning of adolescence and during all the youth period (Davey et al., 2008; Hankin, 2015). Given the functional impairment, suicide risk, and disrupted transition to adulthood associated with depression, as well as its continuity to adulthood (Carballo et al., 2011), it is of major concern to examine factors which may be instrumental to protect youth mental health.

Some authors have argued that youth transition to adulthood now lasts longer than ever before (Sawyer et al., 2018). In this line, emerging adulthood is now defined as a developmental life stage that starts from late adolescence and has been extended to 29 years (Arnett et al., 2014). Thus, youth period has been extended, since educational training now requires more years than ever, and many of our young people need to be enrolled at high school and university to increase their possibilities to find a job. A strength-based conception of youth period could be recommended to protect mental health. Positive youth development (PYD) has shown promise in research on youth resilience and well-being. Lerner et al. (2011) described the relational, developmental systems model of PYD. This model presents adaptive developmental regulations as the mutually beneficial relationships between the individual and the context, which would lead to PYD and some thriving indicators, such as better mental health (Geldhof et al., 2013; Lerner et al., 2015). PYD has demonstrated protective effects on mental health problems (Phelps et al., 2007; Leung et al., 2017; Zhou et al., 2020), as well as positive relations with youth well-being and thriving (Edwards et al., 2007; Gomez-Baya et al., 2019; Kozina et al., 2019), across diverse samples. It has also proven to be a framework suitable for examining cultural differences in youth development (Wiium and Dimitrova, 2019). In this study, we aim to examine the relationship between PYD and depression scores across two cultural contexts, Croatian and Spanish.

### Croatian and Spanish Cultural Contexts

Some studies have found that factors at the country level explain 13.5% of variance in the prevalence of depression and that this variance was increasing with decreasing economic development of countries (Rai et al., 2013). In this study, we have focused on two countries that can be classified as high-income countries compared to many other countries according to the World Bank Rating, but have relatively low GDP compared to the other member states of the European Union (EU). Spain’s GDP per capita is twice as high as Croatia’s, but both are below the average GDP in the European Union (Eurostat, 2020). Slower economic development can reflect in both parents’ and youth unemployment rate, which can increase the risk for mental health problems in youth through multiple paths, including increased stress, parental conflict, divorce, poorer parenting, and decreased support in case of parental unemployment (Agerbo et al., 2001; Fuller-Thomson et al., 2013), as well as increased risk and criminal behaviors in case of youth unemployment (Thern et al., 2017; Mokona et al., 2020). Spain and Croatia are on the top of the list of European countries according to both youth unemployment and unemployment in the general population with rates being twice as high as the European average (European Council, 2017). Moreover, in both Spain and Croatia, youth unemployment doubled the unemployment rate in the general population (European Council, 2017). In spite of the relatively low GDP and the high unemployment rates, depression rates in Croatia and Spain are relatively low, compared to other EU countries. In 2017, 1% of youth aged 15–24 in Croatia reported having chronic depression, while that rate was 1.7% in Spain, and 3.6% among youth in the EU countries on average (Eurostat, 2020). That implies that high unemployment rates and slow economic development do not reflect in the depression rates in youth. Depression rate in Croatia was even lower than in Spain despite the markedly lower GDP. This result points out the need to investigate the potential factors that might protect youth in these countries from developing depression.

Previous research has shown other similarities between youth in Spain and Croatia. For example, equal number of 15-year-olds in Croatia and Spain reported having high life satisfaction (83–84%), feeling low (19%), and having multiple health problems more than once a week (34%) (Inchley et al., 2016). In some studies university students in Spain and Croatia reported comparable (moderate) levels of stress (Cena et al., 2021). Furthermore, there were fewer 15-year-olds who felt that they had high family support, peer support and classmate support in Spain than in Croatia (Inchley et al., 2016). At the same time, an EU survey has shown that Spain has the fewest number of people aged 15 and over who perceive that they have poor social support, and Croatia is not far behind (Eurostat, 2018). Furthermore, 40% of Croatian and 35% of Spanish 15-year-olds reported having good family communication. Moreover, 65% of 15-year-olds in Croatia perceived their school performance as high or very high, compared to around 55% of 15-year-olds in Spain (Inchley et al., 2016). In addition, 60% of the 15-year-old boys and 70% of 15-year-old girls in Spain felt pressured by schoolwork, while that percentage for both boys and girls in Croatia is 39 (Inchley et al., 2016). These aspects of PYD are at least partly influenced by the social environment and cultural values. Therefore, Croatian and Spanish contexts and youth experiences have multiple similarities, which make them suitable for comparing relations between PYD dimensions and depression in youth.

Most studies from PYD framework have been conducted with samples from North America or Northern Europe, so that more evidence is needed to extend the validity of this theory in other countries. In this line, in Croatia and Spain, two countries from the South of Europe, this strength-based conception of transition to adulthood has just begun to reach supportive evidence (Gomez-Baya et al., 2021; Kurtovic et al., 2021). Moreover, more descriptive results are needed in these two countries to guide program design in the respective contexts, and to allow for cross-national comparisons. Despite that prevention programs of depression have been dominated by the reduction of risk factors, the design could be complemented with the development of protective factors (Shoshani and Steinmetz, 2014). PYD theory could be a promising approach to guide research and practice in depression prevention in Croatia and Spain.

### Evidence for the Associations Between Positive Youth Development and Depression

Positive Youth Development represents a view of youth development that shows youth strengths, instead of weaknesses. Accordingly, PYD focuses on strengths that make young people more resistant to negative outcomes, like depression, and more capable to choose a positive life direction (Benson et al., 2006). PYD is proposed to include five dimensions or 5Cs: competence (which refers to a positive perception of efficacy in different domains like social and vocational), confidence (which means an overall positive self-worth), connection (i.e., having positive social relationships), character (i.e., having a sense of integrity and being respectful of the rules of culture and society), and caring (which refers to a sense of empathy and sympathy for others) (Lerner et al., 2015). More research addressing the separate effects by the 5Cs on mental health is recommended, rather than only the effect of overall PYD score.

The comprehensive nature of PYD framework is more suitable for research regarding mental health than just focusing on individual risk factors, because mental health outcomes in youth are multidetermined and the role of protective factors is also prominent (Benson et al., 2006; Zimmerman et al., 2008). Studies so far have showed that PYD could protect youth mental health both directly and indirectly. For example, Leung et al. (2017) found that PYD had direct effects on depression in Dutch and Hong Kong students, but also mediated the relation between childhood maltreatment and depression. Other studies have also demonstrated protective effects of PYD, either overall or individual dimensions, on mental health (Phelps et al., 2007; Olson and Goddard, 2015; Zhou et al., 2020). Strong character has been associated with self-regulation and self-efficacy, which protects against depression (Gardner et al., 2008). Competence have also shown protective effects in terms of protecting against feeling of hopelessness, helplessness and depression (Abela and Hankin, 2009). Furthermore, studies consistently report protective effects of confidence and self-image (Orth and Robins, 2013; Sowislo and Orth, 2013; Bleidorn et al., 2016), as well as social competence and support (Lee et al., 2007; Grav et al., 2012; Camara et al., 2014). In Norway, Holsen et al. (2017) detected negative associations between PYD and depressive symptoms, except for the dimension of caring, which showed a positive correlation. Studies examining the effects of empathy on youth mental health show conflicting results. Some have demonstrated protective effects of empathy due to its association with prosocial behavior and emotional regulation (O’Connor et al., 2007; Rieffe and De Rooij, 2012), while others suggest that more caring youth are more worried about the well-being of their families and more depressed (Zahn-Waxler and Van Hulle, 2011). In China, Zhou et al. (2020) concluded that PYD predicted depression scores after a 1-year follow-up in youth. Milot Travers and Mahalik (2019) indicated that more PYD contributed to lower depression in the United States, but it was especially protective for female subsample. In Hong Kong and the Netherlands, Leung et al. (2017) concluded that PYD factors were protective against depression and suicidal ideation in both cultures. Taken together these results do suggest that PYD could play a crucial role in protection against depression and other mental health problems.

Furthermore, there seem to be multiple developmental trajectories regarding PYD. Zimmerman et al. (2008), using a person-centered approach, identified five distinct trajectories for PYD, thus lending support to the idea that research of youth developmental outcomes needs to take into account the complexities of interindividual differences and intraindividual change, rather than looking at positive and negative development as mutually exclusive. Therefore, studies suggest that the PYD framework provides a wide enough picture of youth development to inform both researchers and practitioners how to promote and protect youth mental health, as well as prevent specific mental health problems. Despite the literature on the relationships between PYD and mental health indicators, like depression, further research is needed to examine the associations between the separate 5Cs of PYD and depression. Moreover, more cross-national evidence is needed regarding the protective effect of PYD on mental health. Thus, examination of these interrelations in countries such as Croatia and Spain contributes to the literature in the research line.

### Gender Differences in Positive Youth Development and Depression

Gender differences in PYD are still not completely clarified. Generally, it is stated that females report higher levels of 5Cs (Phelps et al., 2007; Lerner et al., 2008). Partly contrary, Conway et al. (2015) found that girls reported higher levels of Caring, Character, and Connection, while males reported higher of Confidence and Competence. Ardal et al. (2018) found that females had higher results on Caring, Character, and Connection and males on Confidence, while there were no differences on Competence. The effect size was the largest for Caring. It seems that gender differences are consistent in Caring, Character, and Connection, which is in line with well-known gender differences in importance of and orientation on social relationships (e.g., Rueger et al., 2008). Female also generally show more acceptance of social norms and rules (e.g., Galasso et al., 2020). Advantage of males in Confidence is also in line with previously found gender differences in different variables of self-esteem and self-confidence in young people (e.g., Bleidorn et al., 2016). Results on gender differences on Competence are, however, less clear. Ardal et al. (2018) stated that cultural differences in measurement could have contributed to different results.

There are also gender differences in the rates of depression. Studies consistently show that depression rates are two to three times higher in females than in males (Salk et al., 2017). These differences start to emerge in youth period and continue throughout the life span (Cyranowski et al., 2000). Reasons for this gender gap have been extensively examined, and many explanations have been proposed. They include biological factors (such as hormonal changes in puberty (Steiner et al., 2003), greater exposure to negative life events (Sandanger et al., 2004), including sexual assault (Kendler et al., 2004), gender inequity (Goldston et al., 2008), cognitive factors such as lower self-esteem (Bleidorn et al., 2016) and negative attributional style (Camgoz et al., 2008), body dissatisfaction (Dion et al., 2015), ruminative coping (Krause et al., 2017), greater interpersonal dependence (Cyranowski et al., 2000) as well as conformity to female gender role (Broderick and Korteland, 2002).

Apart from studies which examine gender differences in certain factors which could explain differences in depression, there are also studies which suggest that same factors affect female and male youths differently (Hyde et al., 2008). For example, there are studies suggesting that female youths are more sensitive to problems of significant people in their network, mainly parents and friends (Crawford et al., 2001). Also, there are studies suggesting that girls are more affected by negative events, and that gender differences in appraisal of negative events are greater than differences in the experience of negative events (Sandanger et al., 2004). Apart from gender differences in depression, there are also studies suggesting gender moderations with regard to the effects of certain risk or protective factors. Meadows et al. (2006) demonstrated that girls are more affected by stressful events, with the same level of stress. Also, there are studies suggesting that social support affects mental health more strongly in girls than boys (Davidson and Demaray, 2007; Noret et al., 2020). Calandri et al. (2019) showed that empathy was connected with depression in girls with low emotional self-efficacy. Furthermore, other work has found stronger associations of self-esteem, academic problems and social functioning with depression and anxiety in boys than in girls (Derdikman-Eiron et al., 2012). More research is needed to examine the protective factors’ differential effects by gender (e.g., PYD) on depression.

When we look at gender differences in depression rates in Spain and Croatia, in 2014 depression rates were the same for men in the two countries (5%), but for women the rates were different (Eurostat, 2020). Namely, while 6% of women in Croatia reported that they had chronic depression, the rate in Spain was 11% (Eurostat, 2020). It appears that Spain has higher gender gap in depression rates than Croatia. This cannot be easily explained with the gender inequality index, calculated using data on women’s reproductive health, empowerment, and economic position in the society. In 2013, gender inequality index was higher in Croatia (0.172), than in Spain (0.100) (United Nations Development Programme, 2014). More evidence for the association between youth depressive symptoms and the 5Cs of PYD is needed in other countries from Southern Europe and while controlling for gender differences. In order to develop more efficient prevention programs for depression in youth population, the analysis of how the strengths may protect from depression in each gender could be recommendable.

### Study Aims and Justification

Although some evidence has pointed out the protective effect of PYD on depression, more research is needed to examine the separate effects of the 5Cs of PYD. Moreover, most PYD research to date has studied samples from North America or Northern Europe, and more evidence of the validity of this model is also needed from other countries, such as those from the South of Europe, e.g., Spain and Croatia. As most works in this area have examined samples from a single country, more research is needed to compare the scores on PYD and its effects on mental health in different contexts. Finally, because of the well-supported gender differences in depression among youth in Western countries, the present study is also focused on analyzing the gender differences and the protective effects of PYD on depression.

Thus, the present research has three aims: (a) to describe the 5Cs and depressive symptoms in Croatia and Spain; (b) to examine the relationships between the 5Cs of PYD and depressive symptoms in the Croatian and Spanish sample; and (c) to analyze the gender differences in depressive symptoms and PYD, as well as the gender moderation in the association between both variables. First, we expected to find similar negative association between depression and PYD in both countries, based on previous literature. Second, we hypothesized more depressive symptoms in female youth, but no gender moderation in the relationship between depression and PYD. In line with previous literature, we expected more caring, character and connection in female participants, but less confidence. Third, no differences by country or gender were expected in the interrelations between the 5Cs and depressive symptoms.

## Materials and Methods

### Participants and Procedure

The present research was conducted within the PYD Cross-National Project (Wiium and Dimitrova, 2019). High school students and university students from Eastern Croatia and Southern Spain participated in the study (*M*_age_ = 19.37, *SD* = 2.11). Youth samples were included in the study from both countries to assess a wide age range in the transition to adulthood. There was a total of 584 students from Croatia (207 males, 376 females, one missing an answer about gender; *M*_age_ = 19.19, *SD* = 1.86) and 768 students from Spain (303 male, 465 females; *M*_age_ = 19.50, *SD* = 2.27). Five public high schools (47.9% of the sample of participants) and five public university faculties (52.1% of the sample of participants) of different profiles from Croatia were involved in the study. A total of 45.3% of young people from the Croatian sample have lived in the countryside in the last 10 years, 30.2% in a smaller city, 23.5% in a larger city (Split, Rijeka, Osijek), and 1% in a large city (Zagreb). The majority of high school and university students (until they went to study) lived with both parents (88.4%), 7.9% lived only with their mother, and only with their father 1.2%. Most parents have completed three or 4 years of secondary school (62.3% of fathers and 60.5% of mothers), followed by college or university (27.3% of fathers and 28.6% of mothers) and primary school (5.4% of fathers and 8% of mothers). For 4.8% of fathers and 2.9% of mothers we do not have data on education. In Spain, participants were enrolled in 10 educational institutions located in the region of Andalusia, in both high schools (61.8%) and universities (38.2%). Thus, 43.1% of the sample was enrolled at the last 2 years of high school, 18.7% in professional training, and 38.2% of the sample were taking their first or second year of university studies. Institutions included in Spanish sample were selected by convenience, including different ownership (60% private and 40% public).

Institutional ethics committees approved the study, and written permissions from each school headmaster and faculty dean were obtained. Before completing the questionnaire, participants were informed about the purpose of the study and had the opportunity to sign a written informed consent to participate in the survey. The participation was anonymous and voluntary, with the possibility of abandoning their involvement in the study at any time without consequences. The questionnaires were administered during regular classes. At the beginning of the questionnaire set, there were questions about socio-demographic variables followed by scales measuring indicators of PYD (the 5Cs) and depressive symptoms. These scales assessed different aspects of youth well-being, because PYD measures are focused on strengths and virtues, which are conceived as protective factors for general well-being, while depression questionnaire addresses the presence and intensity of different symptoms, which are indicators of psychological maladjustment.

### Measures

*Positive youth development* (5Cs of PYD-SF; Geldhof et al., 2014). The questionnaire contains 34 items and 5 subscales–Competence (a positive view of one’s actions in different domains; a sample item is *I do very well in my class work at school/university*), Confidence (a sense of self-worth in general, with items such as I am happy with myself most of the time), Character (considered as respect for the rules of one’s society and culture, and a sense of integrity; a sample item is *I hardly ever do things I know I shouldn’t do*), Connection (or positive relationships with others, assessed with items such as *I receive a lot of encouragement at my school/university*), and Caring (defined as developing sympathy and empathy for others; a sample item is *When I see someone being taken advantage of, I want to help them*). Each of the subscales that measure Competence, Confidence, and Caring consist of six items, while the subscales of Character and Connection consist of eight items each. In the Competence, Confidence, and Connections subscales, the participant expresses an opinion on a five-point Likert-type scale where 1 = strongly disagree and 5 = strongly agree. The same form of the Likert-type scale is used for the Character subscale, where 1 = not important and 5 = extremely important. Finally, a five-point Likert-type scale was used for the Caring subscale where 1 = not at all like me and 5 = very much like me. The score on subscales is calculated as sum of responses for each particular subscale. Similar reliability coefficients were shown in the Croatian and Spanish samples. In the Croatian sample, Cronbach’s alphas were between α = 0.67 and α = 0.86 (Competence–α = 0.67, Confidence–α = 0.78, Character–α = 68, Connection–α = 0.77, and Caring–α = 0.86), and in the Spanish sample, the alpha values were between α = 0.63 and α = 0.86 (Competence–α = 0.69, Confidence–α = 0.74, Character–α = 0.83, Connection–α = 0.73, and Caring–α = 0.86). The items were back translated from English by a native professional with training in psychology, ensuring the same meaning as the original English version. Concerning validity, a five-factor structure reached acceptable data fit in confirmatory factor analyses in the present study, χ^2^ (3) = 12.33, *p* = 0.006, CFI = 0.993, RMSEA = 0.050, SRMR = 0.016, after modifications provided by Lagrange multipliers tests (i.e., associations between error of Competence and Confidence, and between errors of Character and Caring).

*Patient Health Questionnaire* (PHQ-9; Kroenke et al., 2001) is a nine-item measure primarily designed to assess depression symptoms (for example, feeling down, depressed, or hopeless, or little interest or pleasure in doing things) according to DSM-IV criteria and for use in primary health care. The nine items correspond to the DSM-IV criteria for Major depressive disorder, and they asses anhedonia, depressed mood, sleep problems, low energy, appetite changes, low self-esteem, concentration difficulties, psychomotor agitation or retardation, and suicide ideation. Participants were asked to estimate how often they were bothered by each symptom over the past 2 weeks on a scale from 0 = almost never to 3 = almost every day. The score is calculated as the sum of all responses on the items. The reliability coefficients were α = 0.84 in the Croatian sample and α = 0.83 in the Spanish sample. Good psychometric properties have been already observed in previous studies in Croatia (Kurtovic et al., 2021) and Spain (Gomez-Baya et al., 2019, 2021).

## Results

### Descriptive Statistics and Configural Invariance

First, descriptive statistics (i.e., mean and standard deviation) of the 5Cs and depression are described in Table 1. In general, moderate to high mean scores in PYD dimensions were observed. The greatest mean in PYD was found for Caring, while the lowest was detected for Competence. Concerning depression, a mean of 8.38 was observed. The mean scores in each depressive symptom (with scores ranging between 0 and 3, depending on severity), ordered by size, were low energy (*M* = 1.53, *SD* = 0.94), sleep problems (*M* = 1.35, *SD* = 1.09), anhedonia (*M* = 1.17, *SD* = 0.81), appetite changes (*M* = 1.15, *SD* = 1.04), depressed mood (*M* = 0.99, *SD* = 0.88), concentration difficulties (*M* = 0.98, *SD* = 0.95), low self-esteem (*M* = 0.79, *SD* = 0.05), psychomotor agitation or retardation (*M* = 0.65, *SD* = 0.89), and suicide ideation (*M* = 0.29, *SD* = 0.69). It should be noted that 18.2% reported having thoughts of suicide several times a week or more. Concerning age, no remarkable associations were observed with any study variable. Only very small interrelations were detected between age and depressive symptoms (*r* = −0.06, *p* = 0.046), and between age and Connection (*r* = 0.07, *p* = 0.018).

**TABLE 1:** Descriptive statistics of depression and PYD, by country and gender.

	Range	Croatia			Spain			Total	Male	Female
		
		Total	Male	Female	Total	Male	Female			
		***M* (*SD*)**	***M* (*SD*)**	***M* (*SD*)**	***M* (*SD*)**	***M* (*SD*)**	***M* (*SD*)**	***M* (*SD*)**	***M* (*SD*)**	***M* (*SD*)**
Competence	1–5	3.38 (0.59)	3.53 (0.63)	3.30 (0.56)	3.24 (0.66)	3.42 (0.64)	3.11 (0.64)	3.30 (0.64)	3.47 (0.64)	3.20 (0.62)
Confidence	1–5	3.90 (0.70)	4.03 (0.66)	3.84 (0.71)	3.69 (0.72)	3.77 (0.68)	3.63 (0.74)	3.78 (0.72)	3.88 (0.68)	3.73 (0.74)
Connection	1–5	3.57 (0.60)	3.51 (0.63)	3.61 (0.58)	3.67 (0.56)	3.57 (0.56)	3.72 (0.55)	3.63 (0.58)	3.55 (0.59)	3.67 (0.57)
Caring	1–5	4.37 (0.67)	3.99 (0.79)	4.57 (0.48)	4.07 (0.71)	3.84 (0.80)	4.22 (0.60)	4.20 (0.71)	3.90 (0.80)	4.38 (0.58)
Character	1–5	3.97 (0.51)	3.82 (0.56)	4.06 (0.47)	3.82 (0.47)	3.71 (0.50)	3.89 (0.44)	3.89 (0.50)	3.75 (0.52)	3.96 (0.46)
Overall PYD	1–5	3.84 (0.40)	3.77 (0.44)	3.88 (0.38)	3.70 (0.40)	3.67 (0.43)	3.72 (0.39)	3.76 (0.41)	3.71 (0.43)	3.79 (0.39)
Depressive symptoms	0–27	8.38 (5.51)	7.86 (5.51)	8.69 (5.49)	9.23 (5.62)	8.38 (5.56)	9.77 (5.60)	8.85 (5.59)	8.16 (5.54)	9.28 (5.57)

Differences by gender and country were examined by performing 2 × 2 variance analyses. For Competence, higher scores were observed in males, *F*(1) = 58.19, *p* < 0.001, η^2^_p_ = 0.042, and in the Croatian sample, *F*(1) = 16.04, *p* < 0.001, η^2^_p_ = 0.012, while no interaction was detected, *F*(1) = 1.36, *p* = 0.243. Males, *F*(1) = 16.01, *p* < 0.001, η^2^_p_ = 0.012, and participants in the Croatian sample, *F*(1) = 31.15, *p* < 0.001, η^2^_p_ = 0.023, showed higher scores in Confidence, with no significant interaction, *F*(1) = 0.41, *p* = 0.525. Regarding Connection, higher mean scores were observed in females, *F*(1) = 15.19, *p* < 0.001, η^2^_p_ = 0.011, and the Spanish sample, *F*(1) = 7.31, *p* = 0.007, η^2^_p_ = 0.005, with no interaction, *F*(1) = 0.41, *p* = 0.525. In the dimension of Character, higher scores were seen in females, *F*(1) = 57.25, *p* < 0.001, η^2^_p_ = 0.042, and among Croatian sample, *F*(1) = 25.50, *p* < 0.001, η^2^_p_ = 0.019, with no significant interaction, *F*(1) = 1.47, *p* = 0.226. Finally, concerning the Caring dimension, a significant interaction between gender and country was detected, *F*(1) = 7.20, *p* = 0.007, η^2^_p_ = 0.005, showing higher scores in Croatian females. In overall PYD, higher score was observed in females, *F*(1) = 10.63, *p* = 0.001, η^2^_p_ = 0.008, and in the Croatian sample, *F*(1) = 30.78, *p* < 0.001, η^2^_p_ = 0.024, with no significant interaction observed, *F*(1) = 1.45, *p* = 0.228. More depressive symptoms were detected in females, *F*(1) = 11.87, *p* = 0.001, η^2^_p_ = 0.009, and in the Spanish sample, *F*(1) = 6.14, *p* = 0.013, η^2^_p_ = 0.005, with no significant interaction.

Furthermore, measurement invariance was analyzed with JASP 0.14.1.0, following the indications by Cheung and Rensvold (2002), to examine whether the overall factor structure for PYD measure shows the same fit by gender and country. Results are presented in Table 2, indicating measurement invariance by gender and significant differences in χ^2^ and CFI by country. The subsequent analyses of the associations with depressive symptoms were conducted using the separate 5Cs of PYD instead of the overall PYD factor.

**TABLE 2:** Measurement invariance by gender and country in the confirmatory factor analysis of PYD measure.

	Model fit			Difference		
	χ ^2^ (df)	*p*	CFI	Δχ ^2^ (Δ df)	*p*	Δ CFI
Gender				−3.55 (0)	0.155	0.008
Male sample	8.80 (3)	0.032	0.989			
Female sample	5.24 (3)	0.155	0.997			
Country				8.59 (0)	0.002	0.010
Croatian sample	6.37 (3)	0.095	0.994			
Spanish sample	14.96 (3)	0.002	0.984			

*CFI = Comparative Fit Index; df = degrees of freedom.*

### Bivariate Correlations by Gender and Country

Table 3 shows the results of the associations between the 5Cs of PYD and depressive symptoms, comparing the results by country and by gender. Differences in the associations were calculated by transforming the correlations to Fisher Z scores, following the indications by Lenhard and Lenhard (2014). Positive associations were observed between the 5Cs, reaching greater magnitude in the interrelations between Confidence and Competence, and between Caring and Character. Negative associations were detected between four dimensions of PYD (i.e., Competence, Confidence, Connection, and Character) and depressive symptoms. Strong correlations were detected between Confidence, Connection and depressive symptoms.

**TABLE 3:** Correlations between the 5Cs of PYD and depressive symptoms by country and gender, and tests of the differences.

																	
	Total sample	Country		Country differences		Gender		Gender differences		Croatia		Gender differences in Croatia		Spain		Gender differences in Spain	
	*n* = 1352	Croatia *n* = 584	Spain *n* = 768	*Z*	*p*	Male *n* = 510	Female *n* = 841	*Z*	*p*	Male *n* = 207	Female *n* = 376	*Z*	*p*	Male *n* = 303	Female *n* = 465	*Z*	*p*
																	
Cp-D	−0.20***	−0.19***	−0.19***	0	0.500	−0.15**	−0.20***	0.92	0.180	–0.13	−0.20***	0.83	0.204	−0.15*	−0.19***	0.56	0.289
Cf-D	−0.44***	−0.46***	−0.41***	–1.12	0.131	−0.40***	−0.45***	1.09	0.139	−0.48***	−0.44***	–0.58	0.280	−0.33***	−0.44***	1.75	0.040
Ch-D	−0.10**	–0.08	−0.09*	0.18	0.427	−0.17***	−0.09*	–1.45	0.074	−0.15*	–0.07	–0.93	0.176	−0.17**	–0.07	–1.37	0.085
Cn-D	−0.31***	−0.31***	−0.32***	0.20	0.420	−0.36***	−0.30***	–1.20	0.116	−0.37***	−0.30***	–0.91	0.182	−0.37***	−0.32***	–0.77	0.222
Ca-D	0.02	0.03	0.05	0.36	0.358	–0.02	–0.01	–0.18	0.429	–0.01	–0.01	0	0.500	–0.02	0.06	–1.08	0.140
Cf-Cp	0.51***	0.52***	0.48***	0.97	0.166	0.52***	0.49***	0.72	0.237	0.54***	0.49***	0.78	0.217	0.50***	0.47***	0.53	0.298
Ch-Cp	0.13***	0.09*	0.14***	–0.92	0.179	0.17***	0.19***	–0.37	0.357	0.07	0.18***	–1.29	0.099	0.24***	0.16**	1.13	0.130
Cn-Cp	0.34***	0.36***	0.34**	0.41	0.339	0.37***	0.37***	0	0.500	0.39***	0.39***	0	0.500	0.37***	0.39***	–0.32	0.376
Ca-Cp	0.09**	0.08	0.06	0.37	0.357	0.19***	0.15***	0.73	0.232	0.20**	0.16**	0.48	0.317	0.17**	0.09	1.10	0.136
Ch-Cf	0.19***	0.16***	0.19***	–0.56	0.287	0.26***	0.20***	1.13	0.130	0.22**	0.18***	0.48	0.316	0.28***	0.17***	1.57	0.059
Cn-Cf	0.43***	0.44***	0.45***	–0.23	0.410	0.46***	0.44***	0.45	0.328	0.53***	0.42***	1.64	0.051	0.43***	0.49***	–1.03	0.152
Ca-Cf	0.08**	0.08	0.03	0.91	0.181	0.17***	0.09**	1.45	0.074	0.16*	0.15***	0.12	0.453	0.15*	–0.01	2.17	0.015
Cn-Ch	0.36***	0.35***	0.40***	–1.06	0.145	0.44***	0.29***	3.09	0.001	0.45***	0.27***	2.39	0.008	0.44***	0.35***	1.44	0.075
Ca-Ch	0.56***	0.53***	0.56***	–0.78	0.219	0.54***	0.53***	0.25	0.402	0.48***	0.52***	–0.61	0.270	0.58***	0.52***	1.16	0.123
Ca-Cn	0.26***	0.30***	0.27***	0.59	0.276	0.30***	0.20***	1.90	0.029	0.35***	0.25***	1.26	0.103	0.27***	0.24***	0.43	0.333

Competence (Cp), Confidence (Cf), Connection (Cn), Caring (Ca), Character (Ch), Depressive symptoms (D). ***p < 0.001; **p < 0.01; *p < 0.05.

No differences in the associations between the 5Cs and depressive symptoms were found by country. Just a few differences were detected by gender. Concerning associations between the 5Cs, Connection presented stronger positive relationship with Character and Caring in the male subsample. In addition, the results indicated that in the Croatian sample, the positive association between Connection and Character was stronger in men. Furthermore, in the Spanish sample, the negative association between Character and depression was only significant in men. The negative effect by Confidence on depression was stronger in female participants. Finally, the positive association between Caring and Confidence was only significant in the male sample (Table 3).

### Hierarchical Regression Analyses

Third, hierarchical regression analyses were conducted to examine overall depression scores, by demographics and the 5Cs, splitting the sample by country and gender. *X*^2^ and *R*^2^ were reported as well as standardized coefficients. Overall PYD was found to be negatively associated with depression scores in both Croatia (*r* = −0.31, *p* < 0.001) and Spain (*r* = −0.29, *p* < 0.001), and in both female (*r* = −0.34, *p* < 0.001) and male (*r* = −0.31, *p* < 0.001) participants. However, different results were observed when examining the separate effects of the 5Cs of PYD. Table 4 shows the results of these analyses.

**TABLE 4:** Hierarchical regression analysis to examine overall score in depression by demographics and 5Cs.

	Total sample			Male			Female		
	*F*	*R* ^2^	β	*F*	*R* ^2^	β	*F*	*R* ^2^	β
**Total sample**	42.86***	0.22		16.91***	0.22		31.79***	0.23	
Country			0.09**			0.06			0.11**
Gender			0.08**						
Age			–0.05			0.02			−0.09**
Competence			0.08*			0.11*			0.06
Confidence			−0.36***			−0.31***			−0.39***
Connection			−0.20***			−0.29***			−0.15***
Caring			0.09**			0.10			0.09*
Character			–0.02			–0.03			–0.01
**Croatia**	24.42***	0.23		10.41***	0.23		18.29***	0.23	
Gender			0.04						
Age			−0.09*			–0.01			−0.15**
Competence			0.10*			0.18*			0.06
Confidence			−0.42***			−0.44***			−0.41***
Connection			−0.17***			−0.24**			−0.12*
Caring			0.10*			0.10			0.08
Character			–0.02			0.01			–0.04
**Spain**	23.92***	0.20		9.66***	0.17		17.70***	0.20	
Gender			0.11**						
Age			–0.02			0.04			–0.06
Competence			0.06			0.08			0.06
Confidence			−0.31***			−0.24**			−0.37***
Connection			−0.23***			−0.31***			−0.17**
Caring			0.09*			0.10			0.09
Character			–0.02			–0.06			0.01

***p < 0.001; **p < 0.01; *p < 0.01.

In the total sample, small effects on depression were detected by gender and country, while no effect was observed by age. Moderate and consistent negative effects were observed by Confidence and Connection on depressive symptoms, while very weak and inconsistent positive effects were detected by Competence and Caring. No remarkable differences were detected among the groups examined. Gender effects were found to be significant in the Spanish sample, while age and country effects were only significant in females. Variance explained was around 22% in the analyses with the whole sample. Thus, these regression analyses underlined the important protective role of Confidence and Connection in depression across genders and countries.

## Discussion

### Differences in Positive Youth Development and Depression by Gender and Country

The aim of our study was to examine the levels of the 5Cs of PYD and depression in Croatian and Spanish youth, as well as their relations and differences across genders and countries. Descriptive values revealed relatively high levels of the 5Cs, overall and individual dimensions in both Croatia and Spanish samples. The highest score was on Caring followed by Character, Confidence, Connection, and Competence, respectively. Other studies across diverse samples have also demonstrated that youths exhibit higher levels of Caring relative to the other PYD dimensions (Gomez-Baya et al., 2019; Kozina et al., 2019). High levels of Caring are consistent with developmental changes in youths regarding the capacity for empathy. Young people become increasingly aware and sensitive to emotions of others, especially people whom they are close with (Allemand et al., 2015; Silke et al., 2018). They also develop idealistic views and sensitivity to perceived injustice (Bondü and Elsner, 2015), all of which could be reflected in the Caring dimension of PYD.

Regarding depression levels, mean value suggests a lower level of overall depression, which is expected given that our sample is non-clinical. The most prevalent symptoms of depression were: low energy, sleep problems, anhedonia, and appetite changes. Of special concern is the finding that over 18% of young people have had thoughts of suicide at least a few times a week. These results, although troubling, are unfortunately consistent with other studies showing an increase in rates of clinically significant levels of depression in youth (Merikangas, 2009; Vicente et al., 2012). We observed some differences in the 5Cs and depression results between Croatia and Spain. Croatian youths reported higher overall PYD scores, as well as more Competence, Confidence, Caring, and Character, while Spanish youths reported more Connection. Furthermore, Spanish youths exhibited higher levels of depression. This is the first study to compare the 5Cs and depression between Croatia and Spain. There are marked similarities between the Croatian and Spanish context of our study; both samples are drawn from a population with a diverse educational background (grammar schools, medical, technical, and vocational schools, as well as faculties of different profiles), both countries are largely Catholic, regions of Croatia and Spain where the study was conducted (Eastern Croatia and Andalusia) are poorer in comparison to other parts of respective countries.

These similarities make our results somewhat surprising. On the other hand, there are indications that youth in Croatia are not encouraged to be socially engaged, responsible for decision making and self-directed, but rather, they are expected to be obedient. While this difference does not explain why Croatian youths appear to exhibit more of the 5Cs and less depression, it does suggest that there may be other societal influences in Spain which might put Spanish youths at an increased risk. For example, in year 2000, Spain had one of the highest response rates on CIDI interview tool for diagnosing current or previous mental disorders, was among the countries with lowest number of psychiatrists per 100,000 inhabitants, and had one of the lowest levels of proneness to seek help for a mental health problem in the EU (European Council, 2017). In 2014, people in Spain sought help for mental health issues more than in Croatia (Eurostat, 2014). In 2017, Spain still had one of the lowest numbers of psychiatrists among the EU countries, much lower than Croatia (Eurostat, 2020). These factors might have contributed to higher levels of depression in Spanish youth. Furthermore, as a protective factor for well-being, according to Eurostat (2020), Spain has the lowest percentage of people aged 15 and over who perceive that they have poor social support and are at the top in the percentage of people with high social support, even better than Croatia. Future research would benefit from detailed examination of structural factors involved in the development of youth depression, as well as resilience and well-being.

We also observed some gender differences in the 5Cs. Girls reported more Connection, Caring, and Character in the whole sample, as well as in the Spanish and Croatian samples, and higher overall PYD scores in Croatia. Globally, female socialization is largely oriented toward investing in and maintaining close relationships, caring for others, and helping behavior. Studies show that throughout youth and well into adulthood, girls and women exhibit more empathy, prosocial behavior, report closer relationships with members of their families, peers and others in their communities (Garaigordobil, 2009; Tsai et al., 2013; Silke et al., 2018). Higher levels of Connection and Caring in girls found in our sample and subsamples are in line with these results. Higher levels of Character in girls observed in our samples is also in line with traditional gender roles, with girls being expected to be more responsible, obedient and respectful of other people and the societal rule, while rebellion against authority and rule breaking is more tolerated in boys (Leaper and Friedman, 2007). The only difference in favor of boys was higher Confidence in the Spanish subsample, which is in line with studies showing greater self-esteem and self-confidence in boys and young men (Bleidorn et al., 2016). This could also be the reflection of socialization influences and pressures female youths experience about social comparison and different opportunities for men and women (Zuckerman et al., 2016). With regard to studies examining gender differences in PYD, our results are in accordance with those demonstrating greater Connection, Caring, and Character in girls across culturally diverse samples (Conway et al., 2015; Ardal et al., 2018; Gomez-Baya et al., 2019). Wiium and Kozina (2021) also found significant gender difference in Caring among youth in Ghana, with girls scoring higher than boys, while Ardal et al. (2018) and Gomez-Baya et al. (2019) also found higher Confidence in boys. However, there are studies suggesting that girls consistently exhibit higher levels of all 5Cs (Lerner et al., 2008), which was not the case in our study. The Croatian sample in our study did show higher overall PYD score in girls, which is in line with Phelps et al. (2007) and Zimmerman et al. (2008). Regarding depression, girls exhibited higher levels of depression, which is consistent with studies demonstrating higher levels of depression in females from youth period throughout adulthood (Kuehner, 2003; Salk et al., 2017). Girls start to experience a rise in symptoms of depression, with estimates reaching up to three times higher as opposed to boys (Hyde et al., 2008), across different cultures (Hopcroft and Burr Bradley, 2007).

### Negative Relationships Between Positive Youth Development and Depression, Controlling for Country and Gender

Our second goal was to examine the associations between the 5Cs and depression. Competence, Confidence, Connection, and Character showed significant negative correlations with depression, while Caring was not significantly correlated with depression. No remarkable differences in the associations were observed by gender or country. This is consistent with other studies demonstrating protective relationships of PYD on youth mental health (Leung et al., 2017; Zhou et al., 2020).

Regression analyses indicated that around 20% of the variance of depressive symptoms could be explained by on the 5Cs. In regression analyses, Confidence and Connection had negative effects on depression in the whole sample, as well as in the Croatian, Spanish, female and male subsamples. Young people who are satisfied with their own capabilities, appearance, and behavior, who think they can tackle challenges and expect positive experiences in the future are likely to be protected from developing depressive symptoms even in light of adversity, because of documented protective effects of self-esteem, self-efficacy, and optimism (Orth and Robins, 2013; Sowislo and Orth, 2013; Kleiman et al., 2017). Connection, on the other hand, reflects not only social support, which has well documented protective interrelations, but also pertains to feeling of being valued and appreciated by others, all of which can protect against depression (Benson et al., 2006; Lee et al., 2007; Grav et al., 2012; Camara et al., 2014). Even more, relationships in which young people feel valued, encouraged and safe present healthy models for the development of future relationships, which can have long-term protective effects (Cawnthorpe et al., 2004).

Even though Competence and Character were significantly correlated with depression, their effects on depression were not significant. More precisely, Competence only showed a suppression effect because of its association with Confidence. While significant correlations of Competence and Character with depression are consistent with other studies (Gable and Shean, 2000; Edwards et al., 2007; Garcia et al., 2012), our results suggest a rather weak association with depression, which fails to demonstrate possible protective effects. Furthermore, despite the negative interrelation between overall PYD and depression, the dimension of Caring had a positive significant effect on depression. There are studies showing protective effects of empathy for mental health in youth (Rieffe and De Rooij, 2012), but there are also studies demonstrating that increased sensitivity to emotions of others can make youths more vulnerable if they do not have sufficient support and possess good emotional regulation skills (Zahn-Waxler and Van Hulle, 2011). Calandri et al. (2019) found that the effects of youth empathy on depression were moderated by parental support, in a way that empathy posed a risk in the absence of parental support. This paradoxical effect by caring on mental health was already noted by previous research (Holsen et al., 2017). Geldhof et al. (2019) showed that Caring was positively associated with depression, concluding that “young people may suffer if they care too much” (p.1) and this dimension should grow adaptive developmental regulations with the context in order to lead to positive outcomes. In this same line, Caring was found to be positively related to other problems in mental health, such as anxiety (Kozina et al., 2021). Moreover, the dimension of Character, defined as respect for the rules of the society, is strongly and positively correlated with Caring. Character and depression were not found to be significantly interrelated in the regression analysis, because this dimension of PYD could also need other personal skills (i.e., coping strategies) and external resources (i.e., social support) to produce positive outcomes for health and well-being, as may be derived from previous research (Albanesi et al., 2007; Gestsdóttir and Lerner, 2007; Kadir and Mohd, 2021).

The differences in socialization of females and males might be a pathway which puts the former at an increased risk for depression because it does not facilitate the development of adequate levels of Confidence. Zahn–Waxler et al. (2000) found that empathy for a distressed caregiver can lead to the feeling of responsibility and guilt in young children, serving as an early pathway to internalized problems. They also found that caring for others and self-distress co-occur more often and are more prevalent for girls. This can be true for our sample, in a way that females, who lack faith in their abilities to cope with challenges, might be additionally burdened by both societal expectation and their own capacity for empathy. Furthermore, our results suggest that the effect of Confidence on depression was stronger among Croatian youths, who reported higher average levels of Confidence than Spanish youths.

### Limitations

There are some limitations of our study, which should be noted. First, the correlational and cross-sectional design prevents us from making causal conclusions, as well as conclusions regarding whether the 5Cs temporally precede depressive symptoms. Studies with a longitudinal design and spanning over a longer period may take us a step further in predicting the direction of the relationship between the 5Cs and depression. In addition, self-report measures, especially for depression, can be under the influence of emotional states and socially desirable answering. However, self-report of youth mental health outcomes has been found to be quite reliable (Ridge et al., 2009). Future research should use a multi-method approach and include an instrument to assess depression more comprehensively. Moreover, it is possible that our convenience sampling method affected the results. Although high school students are representative of the high school populations in Croatia and Spain, university students may not be since they are already pre-selected. Future studies should also involve young people not attending university.

Finally, although the 5Cs are largely reported among our Croatian and Spanish youths, there is still the question of how well these Cs capture the positive outcomes and thriving indicators. Consistent with Geldhof et al. (2014), the 5Cs measures that were created to capture broad aspects of positive development among most young people, may lack content specificity for other groups of youth. Thus, the meaning of Competence, Confidence, Connection, Caring, and Character may differ across contexts. These are issues that can be resolved in future research, utilizing a combination of quantitative and qualitative methods to assess indicators that truly depict positive development of Croatian and Spanish youth.

### Implications for Practice

The results of our study have important implications. First, promotion of the 5Cs of PYD could be a meaningful and important approach to protect youth mental health by preventing depressive symptoms. Given the fact that depression is multidetermined, and the emphasis is on the number of risk factors rather than their quality (Cicchetti and Toth, 1998), focusing on building resilience on a wider scale with strength-based strategies, can be more fruitful than targeting specific risk or protective factors, such as self-esteem, coping or parenting. Second, PYD is a community wide approach, meaning that all domains should be involved, including family systems, schools, neighborhoods, and a wider community. PYD intervention programs have already showed effectiveness in other countries (e.g., EEUU; Catalano et al., 2004) to protect health and psychological well-being in youth samples by developing the 5Cs. Research evidence described in the present work may support the design of practices in Croatia and Spain to foster the 5Cs of PYD in order to prevent depression from a gender perspective. Third, experts working with clinical populations should employ strategies that promote healthy and empowering relationships, as well as building confidence, with specific actions for female youth.

## Conclusion

This study has examined the 5Cs of PYD and depressive symptoms in Croatia and Spain, as well as their associations and gender differences. Some contributions have been provided. Some gender differences were detected in PYD dimensions and depressive symptoms, showing that boys had more Competence and Confidence, while girls presented more Connection, Caring and Character, as well as more depressive symptoms. Regarding the relationships between PYD indicators and depressive symptoms, the dimensions of Confidence and Connection were significant main effects, showing that more Confidence and more Connection were related to fewer depressive symptoms. No remarkable differences by gender or country were detected in the associations between the 5Cs and depressive symptoms. With the current findings, youth mental health services and initiatives that engage the partnership of youth contexts, such as the family, schools, neighborhoods, and the wider community should create a more inclusive and sustainable support for the development and protection of youth well-being.

## Data Availability Statement

The raw data supporting the conclusions of this article will be made available by the authors, without undue reservation.

## Ethics Statement

The studies involving human participants were reviewed and approved by University of Huelva; Josip Juraj Strossmayer University of Osijek; and NSD – Norwegian Centre for Research Data at the University of Bergen (approval number-51708/3/IJJ). The patients/participants provided their written informed consent to participate in this study.

## Author Contributions

All authors listed have made a substantial, direct, and intellectual contribution to the work, and approved it for publication.

## Conflict of Interest

The authors declare that the research was conducted in the absence of any commercial or financial relationships that could be construed as a potential conflict of interest.

## Publisher’s Note

All claims expressed in this article are solely those of the authors and do not necessarily represent those of their affiliated organizations, or those of the publisher, the editors and the reviewers. Any product that may be evaluated in this article, or claim that may be made by its manufacturer, is not guaranteed or endorsed by the publisher.
